# Supplementary material for: Orthogonal spectral and temporal envelope representation during the onset phase in auditory cortex
Source: iScience. 2025 Oct 17;28(11):113796. doi: 10.1016/j.isci.2025.113796 (PMC12630032; doi:10.1016/j.isci.2025.113796)
Supplement: Document S1. Figures S1–S4 [file mmc1.pdf]

iScience, Volume *28*

Supplemental information

Orthogonal spectral and temporal envelope representation during the onset phase in auditory cortex

Kuniyuki Takahashi, Tianrui Guo, Tatsuya Yamagishi, Shinsuke Ohshima, Hiroaki Tsukano, and Arata Horii

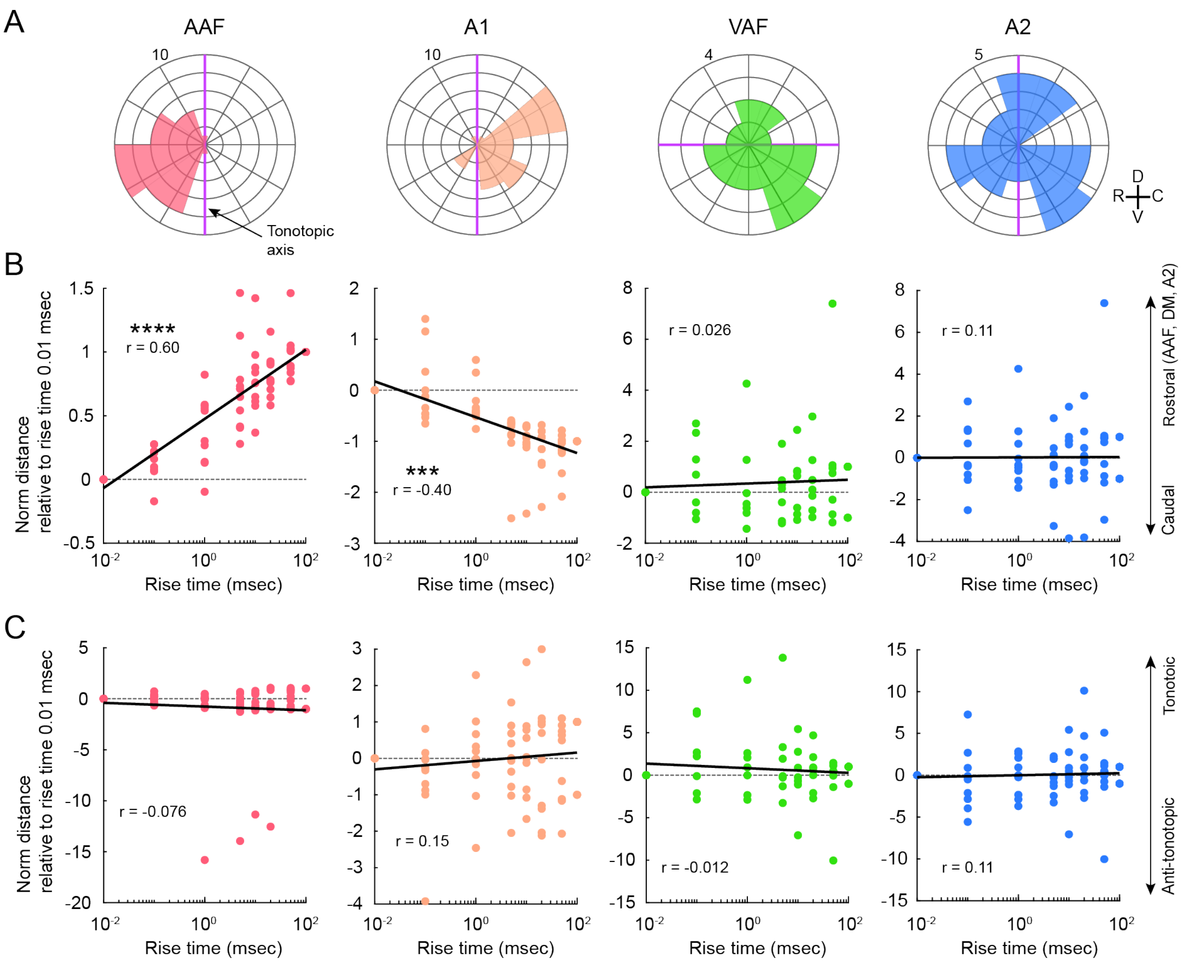

Figure S1. Envelope maps in left auditory cortex.

(A) Circular histogram showing the direction of responses at rise-ramp times of 100 msec relative to 0.01 msec in the left auditory cortical areas. Purple lines indicate the tonotopic axis. Data for 5, 20, 40 kHz are co-plotted. AAF, n = 30 plots; A1, n = 30 plots; VAF, n = 26 plots; A2, n = 30 plots from 10 mice. **(B)** Relationship between rise-ramp time and the shift from the peak location for a rise-ramp time of 0.01 msec in the direction orthogonal to tonotopy. AAF, r = 0.60, ****p = 3.9 × 10^{-9}, n = 80 plots (Pearson's correlation); A1, r = -0.40, ***p = 2.1 × 10^{-4}, n = 80 plots; VAF, r = 0.026, p = 0.83, n = 72 plots; A2, r = 0.11, p = 0.33, n = 80 plots. **(C)** Relationship between rise-ramp time and the shift from the peak location for rise-ramp time of 0.01 msec in the tonotopic direction. AAF, r = -0.076, p = 0.50, n = 80 plots; A1, r = 0.15, p = 0.19, n = 80 plots; VAF, r = -0.012, p = 0.92, n = 72 plots; A2, r = 0.11, p = 0.35, n = 80 plots.

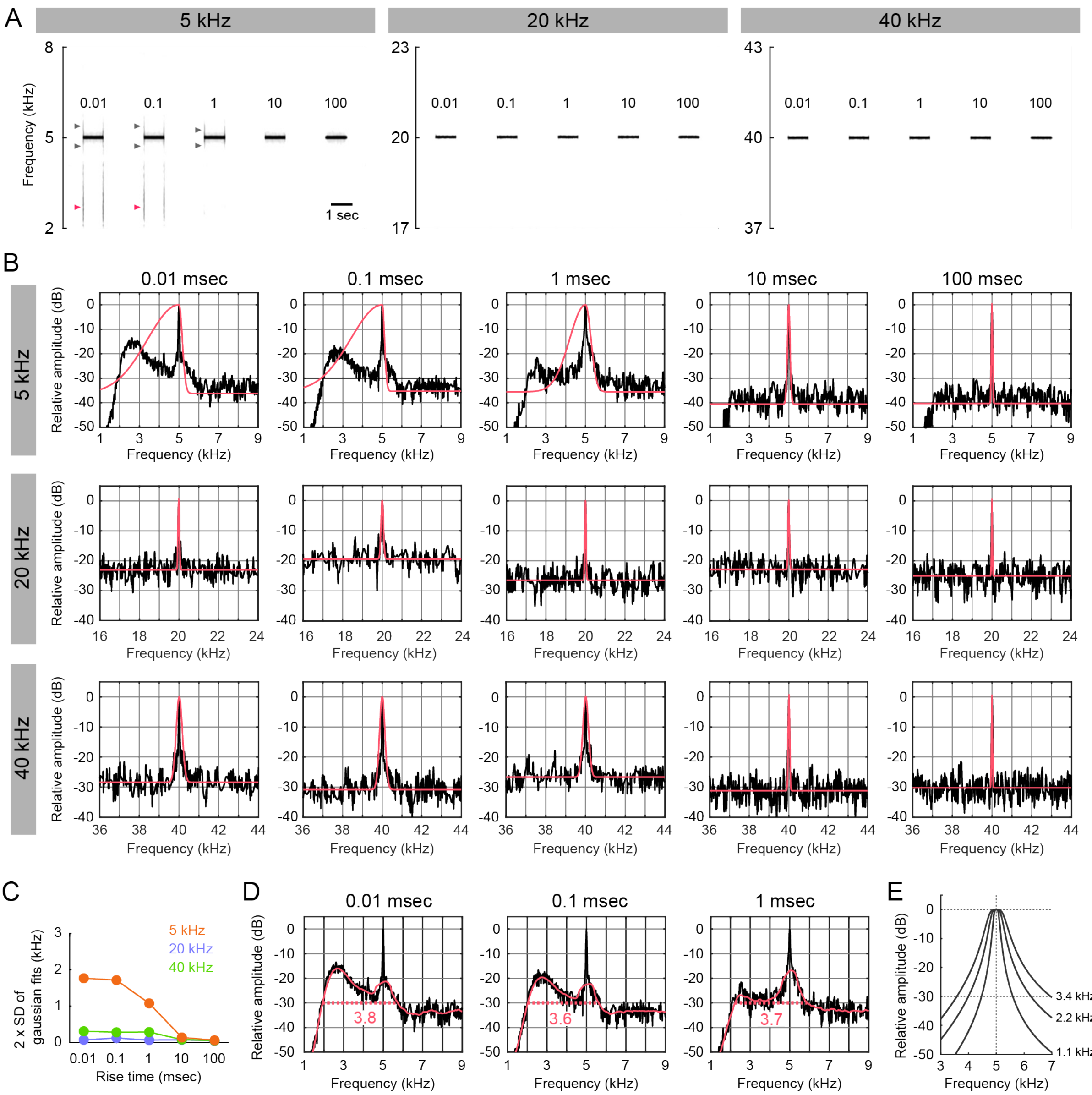

Figure S2. Spectral analysis of pure tones with varying rise times.

(A) Spectrograms of 5, 20, and 40 kHz pure tones with rise times ranging from 0.01 to 100 msec. Gray arrowheads indicate mild, symmetrical spectral splatter around the carrier frequency, and red arrowheads indicate broader, lower-frequency–biased splatter. **(B)** Amplitude spectra at tone onset across carrier frequencies and rise times. Gaussian fits are shown in red. Y-axis values are expressed in dB relative to the peak amplitude at the carrier frequency. **(C)** Frequency ranges covering ±2 SD of the Gaussian fits shown in (B). **(D)** Additional quantification of splatter width. Red curves represent polynomial fits, and red values and dotted lines indicate the width at –30 dB. In (B), Gaussian fits were used to estimate splatter width, but fitting accuracy was limited for 5 kHz tones with rise times of 0.01, 0.1, and 1 msec. Therefore, polynomial fitting was also applied to these data to re-evaluate splatter width, yielding values similar to those obtained with Gaussian fitting shown in Figure 5C. **(E)** Band-pass noise curves added to 5 kHz pure tones. Values indicate the width of band-pass noise at –30 dB, matching the frequency range of splatter shown in Figure 5C.

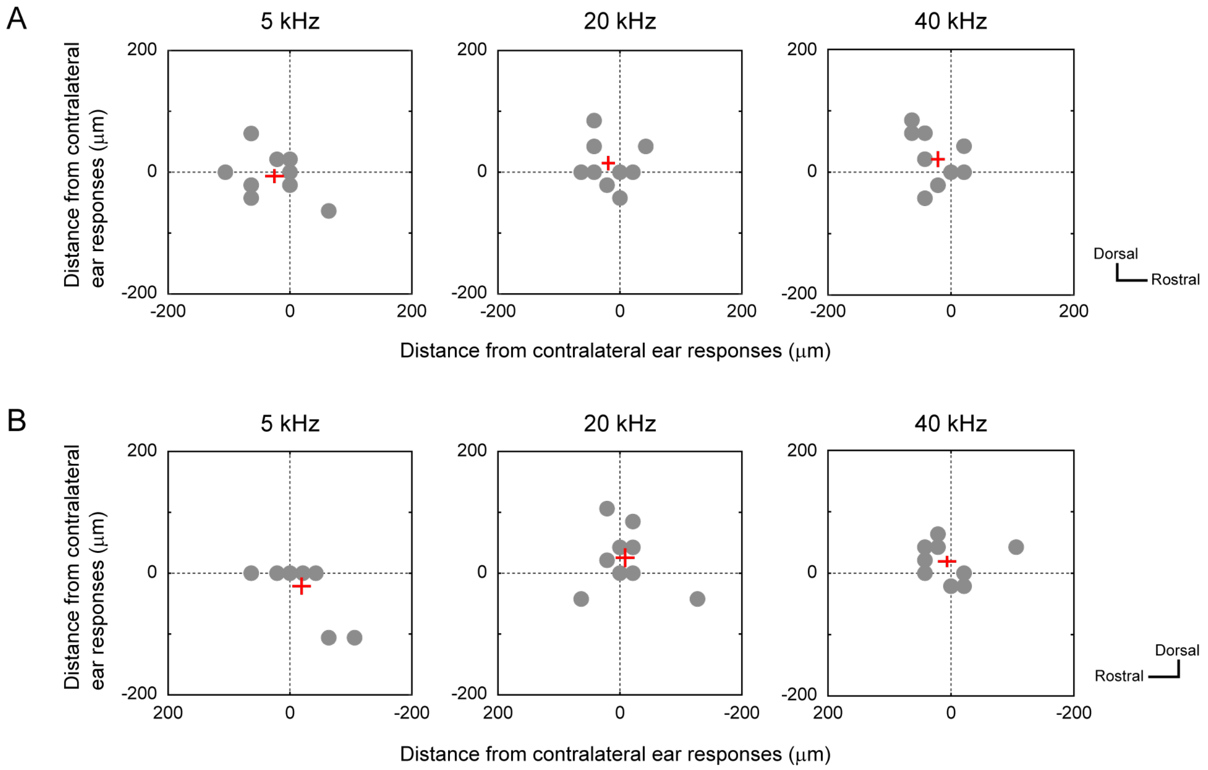

Figure S3. No difference in response locations between contra- and ipsilateral sound inputs.

(A) Deviation of AAF responses elicited by ipsilateral ear stimulation relative to those elicited by contralateral stimulation in the right hemisphere of the same mice. Results for 5, 20, and 40 kHz at a 10-msec rise time are shown separately. Gray dots indicate each data. Red crosses indicate mean ± SEM. No significant differences were observed along either the horizontal or ventral axis at any frequency (two-sided paired t-test, $p > 0.05$, $n = 9$ mice). **(B)** Same as (A), but for AAF responses in the left hemisphere. No significant differences were observed along either axis at any frequency ($p > 0.05$, $n = 9$ mice).

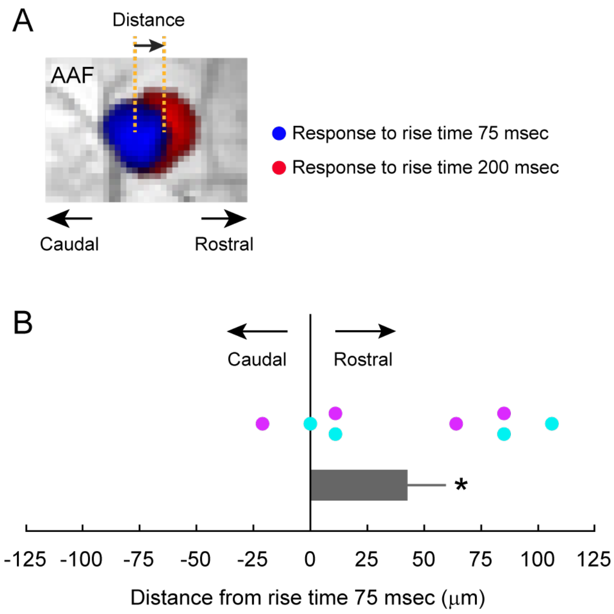

Figure S4. Envelope maps during the ramping phase.
(A) Explanation of analysis. Positional shifts of 5 kHz responses are evaluated between rise times of 75 and 200 msec. **(B)** Plots of rostro-caudal deviation between the peak responses for a rise-ramp time of 200 msec relative to that for a rise-ramp time of 75 msec in AAF. Positive values indicate that the response for a rise-ramp time of 200 msec is rostral to that for 75 msec, while negative values indicate it is caudal. Data for AAF in the right (cyan) and left hemisphere (purple) are co-plotted (n = 8 plots from 4 mice). Data are shown in mean ± SEM. *p = 0.039 (two-sided paired t-test).
